# New Medical Journals

**Published:** 1859-02

**Authors:** 


### NEW MEDICAL JOURNALS.

In the last few weeks we have received *four* new Medical Journals, *two* from Louisville, Kentucky—"The Semi-Monthly Medical News," edited by S. M. Bemiss, M. D., Professor of Clinical Medicine, University of Louisville; J. W. Benson, M. D., Professor of Descriptive and Surgical Anatomy, University of Louisville. And in addition from the same city, "The Louisville Medical Gazette, by L. J. Frazee, M. D., (Semi-monthly.) We next have the Saint Joseph Journal of Medicine and Surgery, a Bi-Monthly Journal, edited by J. H. Crane, M. D., O. B. Knode, M. D., and G. C. Catlett, M. D., an Editorial Committee for the St. Joseph Missouri Medical Society, under whose auspices and supervision it is published.

And last though not by any means least in our estimation, we acknowledge the receipt of "The New York Medical Press," a weekly Journal of Medicine, Surgery, and the collateral sciences, Edited by J. L. Kiernan, A. B., M. D., and W. O'Meagher, M. D.

These Journals all seem to breathe the right spirit, and we cordially extend to them the right hand of fellowship, and with pleasure place them upon our exchange list.

As soon as we complete the fortune we are so rapidly acquiring, as a Medical Editor, it is our intention to retire, for the benefit of some one of the numerous hungry brethren, who are on the lookout for a lucrative position.

